# Household access to basic drinking water, sanitation and hygiene facilities: secondary analysis of data from the demographic and health survey V, 2017–2018

**DOI:** 10.1186/s12889-022-13665-0

**Published:** 2022-07-14

**Authors:** Nicolas Gaffan, Alphonse Kpozèhouen, Cyriaque Dégbey, Yolaine Glèlè Ahanhanzo, Romain Glèlè Kakaï, Roger Salamon

**Affiliations:** 1 Department of Epidemiology and Biostatistics, Regional Institute of Public Health, University of Abomey-Calavi, Ouidah, Benin; 2 Department of Environmental Health, Regional Institute of Public Health, University of Abomey Calavi, Ouidah, Benin; 3 University Hospital Hygiene Clinic, National Hospital and University Centre Hubert Koutoukou Maga, Cotonou, Benin; 4 Laboratory of Biomathematics and Forest Estimations, University of Abomey-Calavi, Cotonou, Benin; 5 Institute of Public Health, Epidemiology and Development, Victor Segalen University, Bordeaux, France

**Keywords:** Determinant, Logistic regression, Household, Access, Water, Sanitation, Hygiene, Map, National data, Benin

## Abstract

**Background:**

In Benin, access to water, sanitation and hygiene (WASH) remains an issue. This study aims to provide an overview of household access to basic WASH services based on nationally representative data.

**Method:**

Secondary analyses were run using the ‘HOUSEHOLD’ dataset of the fifth Demographic and Health Survey 2017–2018. The dependent variables were household access to individual and combined basic WASH services. The characteristics of the household head and those related to the composition, wealth and environment of the household were independent variables. After a descriptive analysis of all study variables, multivariate logistic regression was performed to identify predictors of outcome variables.

**Results:**

The study included 14,156 households. Of these, 63.98% (95% CI = 61.63–66.26), 13.28% (95% CI = 12.10–14.57) and 10.11% (95% CI = 9.19–11.11) had access to individual basic water, sanitation and hygiene facilities, respectively. Also, 3% (95% CI = 2.53–3.56) of households had access to combined basic WASH services. Overall, the richest households and few, and those headed by people aged 30 and over, female and with higher levels of education, were the most likely to have access to individual and combined basic WASH services. In addition, disparities based on the department of residence were observed.

**Conclusion:**

The authors suggest a multifactorial approach that addresses the identified determinants.

## Background

In 2010, the United Nations General Assembly (UNGA) recognised the right to drinking water and sanitation as a human right and called on states to intensify efforts to provide safe, clean, accessible and affordable drinking water and sanitation for all [1]. Also, in 2015, the Member States of the United Nations adopted the 2030 Agenda for Sustainable Development, Goal 6 of which aims to “ensure availability and sustainable management of water and sanitation for all” [2].

In 2020, 489 million people worldwide still lacked access to improved drinking water facilities—water points that can deliver safe water because of their design and construction—including 122 million people using surface water (river, dam, lake, pond, stream, canal or irrigation canal) for drinking water [3, 4]. People's access to improved sanitation facilities—facilities designed to hygienically separate excreta from human contact—increased over 2000–2020 [3, 4]. However, in 2020, 494 million people were still practising open defecation [3]. In addition, 670 million people do not have handwashing facilities with soap and water [3]. Evidence shows that contaminated water and poor sanitation are associated with the transmission of diseases and other symptoms such as cholera, bacillary diarrhoea, viral hepatitis A, typhoid, polio and acute respiratory infections, etc. [5–11]. According to the World Health Organization (WHO), inadequate access to Water, Sanitation and Hygiene (WASH) services is responsible for nearly 2 million deaths annually worldwide, most of them children [11]. Sub-Saharan Africa still has the largest burden of morbidity and mortality due to inadequate WASH facilities (60% and 53% of all DALYs and deaths attributable to inadequate WASH facilities, respectively) [11].

In Benin, access to appropriate WASH facilities remains an issue. In its Health Development Plan (PNDS, *Plan National de Développement Sanitaire* in French) 2018–2022, Benin defined the promotion of hygiene and basic sanitation as a key action to prevent and fight diseases [12]. Therefore, Objective 6 of the National Development Plan (PND) 2018–2025, which guides the government's actions, aims “to guarantee access for all to water supply and sanitation services” [13]. In addition, in 2018, Benin adopted the National Strategy for the Promotion of Hygiene and Basic Sanitation (SNPHAB*, Stratégie Nationale de Promotion de l’Hygiène et de l’Assainissement de Base* in French) in rural areas [14]. This 12-year strategy (2018–2030) aims to “ensure equitable access to adequate sanitation and hygiene services for the rural population of Benin” [14]. Furthermore, like several other low-income countries, Benin benefits from the technical and financial assistance of several partners to improve people's access to WASH services. In particular, the United Nations International Children's Fund (UNICEF) is implementing the Community-Led Total Sanitation (CLTS) approach, which aims to support and encourage communities to take collective action to improve their hygiene and sanitation practices [15–17]. However, the high morbidity and mortality indicators for waterborne diseases show that there are still significant gaps in people's access to appropriate WASH services. In Benin, 13,390 (14%) deaths and 1,028,459 (15%) DALYs are attributable to inadequate WASH facilities in 2016 [18]. Also, ten children continue to die every day, 90% of these deaths being because of the ingestion of contaminated water and the lack of community sanitation facilities [16]. Specifically, the prevalence of diarrhoeal diseases was 11%, with a case fatality rate of 16 deaths per 10,000 children [19, 20].

Consequently, efforts to improve access to appropriate WASH services are required. For these interventions to be successful, the surveillance of progress in coverage of WASH services needs to be enhanced, and the inequalities that determine household access to these facilities need to be better understood. According to studies in Africa and Asia, the factors associated with household access to improved or basic WASH services were the characteristics of the household head and the composition, wealth and environment of the household [21–31]. So far, in Benin, there is scarce information on disparities in people's access to WASH facilities. One relevant study highlighted socio-demographic and environmental factors but was limited to a specific geographical area (the commune of Lalo) [32]. However, the national coverage of WASH services is regularly monitored every five years through the Demographic and Health Surveys (DHS). To date, Benin has conducted five DHS. The results of the Fourth Demographic and Health Survey (DHS-IV) showed that despite progress in terms of household access to improved drinking water sources, the use of water from unprotected wells is still widespread (15%), with 3.6% of households using surface water for drinking water [33]. In addition, nearly two-thirds of households (66.4%) had access to unimproved toilets, and 54.2% did not have any sanitation facilities [33]. Also, 43% of households did not have a handwashing facility [33].

In 2017–2018, the Fifth Demographic and Health Survey (DHS-V) took place and provided data on the coverage of households with WASH facilities. Thus, the present work aims to study household access to WASH facilities based on nationally representative data of the Beninese population collected during the DHS-V.

## Methods

### Study area

Benin is a West African state covering an area of 114,763 km^2^ with an urbanization rate of 44% [34]. The Fourth General Census of Population and Housing (*Recensement Général de la Population et de l'Habitation* in French, RGPH-IV) in 2013 counted 10,008,749 inhabitants, 51.2% of whom were women [35]. According to estimates, the population growth is about + 2.7% per year [34]. The 2019 projections put the population in Benin at 11,884,127 (5,846,550 men and 6,037,577 women) [34]. Administratively, Benin has 12 departments divided into 77 communes.

### Study design and data source

This study used a cross-sectional design and consisted of a secondary analysis of data obtained from the DHS-V. The DHS surveys are a standard series of surveys (DHS-I in 1996, DHS-II in 2001, DHS-III in 2006, DHS-IV in 2011–2012 and DHS-V in 2017–2018) at the national level that provide up-to-date estimates of basic demographic and health indicators. The DHS-V was conducted by the National Institute of Statistics and Demography (INStaD, *Institut National de la Statistique et de la Démographie* in French) in collaboration with the Ministry of Health and with technical support from ICF through the DHS Program of the United States Agency for International Development (USAID). Details on the DHS Program are described elsewhere [36]. In this study, the unit of analysis was households. Following a request sent via the DHS Program website—https://dhsprogram.com/—DHS-V ‘HOUSEHOLD’ dataset (BJHR71DT) was downloaded.

### Sampling procedure and sample size

The DHS-V employed a nationally representative sample of the Beninese population using a two-stage stratified sampling procedure. The twelve departments were stratified into urban and rural areas, except for Littoral, an entirely urban stratum. This stratification resulted in 23 strata. In each stratum, a specific number of Primary Sample Units (PSUs) were systematically selected (in the first stage) with Probability Proportional to the Size (PPS). The list of Enumeration Areas (EAs) established during the RGPH-IV served as the sampling frame for this selection. After listing the households within the selected EAs, a systematic sample of 26 households was drawn from each PSU (in the second stage). Details on the survey sampling procedure and data collection methods are described elsewhere [37]. Of the 14,435 households selected, 14,293 were identified during the survey [37]. Of these, 14,156 (response rate = 99%) were successfully surveyed [37].

### Study variables

#### Dependent variables

The dependent variables were household access to basic WASH services. By the WHO/UNICEF Joint Monitoring Programme (JMP) guidelines, household access to a source of drinking water, sanitation, and hygiene could be grouped according to the level of service provided: “basic”, “limited”, “unimproved” and “no service” (Tables 1 and 2) [4]. A dichotomisation was performed to obtain the dependent variables: yes = 1 when the service level was basic, and no = 0 otherwise (individual basic WASH services). Finally, a last binary dependent variable was generated for the households that combined all three basic facilities (combined basic WASH services).

**Table 1:** WHO/UNICEF Joint Monitoring Programme (JMP) ladder for water, sanitation and hygiene (WASH) services

Service level	Water	Sanitation	Hygiene
Basic	Drinking water from an improved source, provided collection time is not more than 30 min for a round trip, including queuing	Use of improved facilities that are not shared with other households	Availability of a handwashing facility on premises with soap and water
Limited	Drinking water from an improved source for which collection time exceeds 30 min for a round trip, including queuing	Use of improved facilities shared between two or more households	Availability of a handwashing facility on premises without soap and water
Unimproved	Drinking water from an unprotected dug well or unprotected spring	Use of pit latrines without a slab or platform, hanging latrines or bucket latrines	Not applicable
No service	Surface water	Open defecation	No handwashing facility on premises

**Source** Adapted from WHO; UNICEF. Progress on Drinking Water, Sanitation and Hygiene: 2017 Update and SDG Baselines; WHO: Geneva, 2017; ISBN 978–92-4–151,289-3. [4]

**Table 2:** JMP classification of improved/unimproved water and sanitation facility types

Facility types	Water	Sanitation
Improved facilities	Piped supplies • Tap water in the dwelling, yard or plot • Public standposts Non-piped supplies • Boreholes/tubewells • Protected wells and springs • Rainwater • Packaged water, including bottled water and sachet water • Delivered water, including tanker trucks and small carts	Networked sanitation • Flush and pour flush toilets connected to sewers On-site sanitation • Flush and pour flush toilets or latrines connected to septic tanks or pits • Ventilated improved pit latrines • Pit latrines with slabs • Composting toilets, including twin pit latrines and container-based systems
Unimproved facilities	Non-piped supplies • Unprotected wells and springs	On-site sanitation • Pit latrines without slabs • Hanging latrines • Bucket latrines

**Source** Adapted from WHO; UNICEF. Progress on Drinking Water, Sanitation and Hygiene: 2017 Update and SDG Baselines; WHO: Geneva, 2017; ISBN 978–92-4–151,289-3. [4]

#### Covariates

The independent variables were:

- the variables related to the household head: age (< 30, 30–39, 40–49, 50–59, ≥ 60), sex (male, female), level of education (no formal education, primary, secondary, higher) and marital status (single, in couple);
- the variables related to household's composition and wealth: household size (≤ 5, > 5), children aged five and under in the household (yes, no) and wealth index (poorest, poorer, middle, richer, richest);
- the variables related to the household's environment of residence: area (urban, rural) and department (Alibori, Atacora, Atlantic, Borgou, Collines, Couffo, Donga, Littoral, Mono, Ouémé, Plateau and Zou).

These variables were chosen from a literature review [22, 23, 25, 27, 28, 30]. 

### Data analysis

All analyses included the sample weight. The independent and dependent variables were described by calculating the numbers and percentages of their categories. Also, the spatial distribution of household access to individual and combined basic WASH facilities was described using QGIS 2.18. Chi-square tests were performed to determine the association between the independent and dependent variables. Multivariate logistic regressions were performed to identify predictors of access to individual and combined basic WASH facilities. Potential factors were selected at p < 0.20 using simple logistic regression [38]. They were then entered into a multivariate logistic regression using a backward stepwise strategy to obtain adjusted estimates. For each regression, the indicators used to measure the association between the dependent and independent variables were the odds ratio (OR) and the 95% CI. The significance level was 5%. All statistical analyses were conducted in Stata 15 (StataCorp, College Station, TX, USA).

### Ethical approval

All methods were performed by the principles of the Declaration of Helsinki. Firstly, the launch of the DHS-V data collection was conditional on the authorisation of the National Statistical Council (*Conseil National de la Statistique* in French, CNS) to obtain the statistical visa of opportunity and conformity, and on the approval of the National Committee on Health Research Ethics (*Comité National d’Ethique pour la Recherche en Santé* in French, CNERS) to get the binding scientific and ethical opinion of the survey [37]. These two institutions reviewed and approved the methodological and financial documents and the collection tools [37]. Then, during data collection, the informed consent of eligible respondents was sought before starting the interviews. Finally, the dataset used for the secondary analyses in this study was fully anonymised so that the individuals surveyed could not be identified in any way [37].

## Results

### Basic characteristics of households

The study included 14,156 households. Table 3 presents the basic characteristics of the surveyed households. The majority of household heads were 30–39 years old (26.91%), male (75.12%), and in a couple (77.62%). More than half (53,35%) of the household heads had no formal education. The poorest wealth quintile comprises about 17.67% of the sample compared to 22.82% for the richest quintile. In addition, 61.66% of households had five or fewer members. Children aged five and under were present in 60.45% of households. About 57% of the households lived in rural areas. As regards department of residence, households in Atlantique (13.91%), Ouémé (11.53%) and Borgou (10.58%) were the most represented.

**Table 3:** Basic characteristics of the households in Benin, 2017–2018

Variables	n	%
**Age (years)**		
< 30	2,454	17.34
30–39	3,810	26.91
40–49	2,970	20.98
50–59	2,146	15.16
≥ 60	2,775	19.60
**Sex**		
Male	10,634	75.12
Female	3,522	24.88
**Level of education**		
No formal education	7,553	53.35
Primary	3,232	22.83
Secondary	2,593	18.32
Higher	778	5.50
**Marital status**		
Single	3,169	22.38
In couple	10,987	77.62
**Wealth index**		
Poorest	2,501	17.67
Poorer	2,675	18.89
Middle	2,798	19.77
Richer	2,951	20.85
Richest	3,231	22.82
**Household size**		
≤ 5	8,728	61.66
> 5	5,428	38.34
**Children aged 5 and under in the household**		
No	5,598	39.55
Yes	8,558	60.45
**Area**		
Urban	6,104	43.12
Rural	8,052	56.88
**Department**		
Alibori	1,192	8.42
Atacora	923	6.52
Atlantique	1,969	13.91
Borgou	1,498	10.58
Collines	981	6.93
Couffo	1,108	7.83
Donga	740	5.23
Littoral	852	6.02
Mono	879	6.21
Ouémé	1,633	11.53
Plateau	984	6.95
Zou	1,399	9.88

### Household access to WASH services

**Fig. 1:**
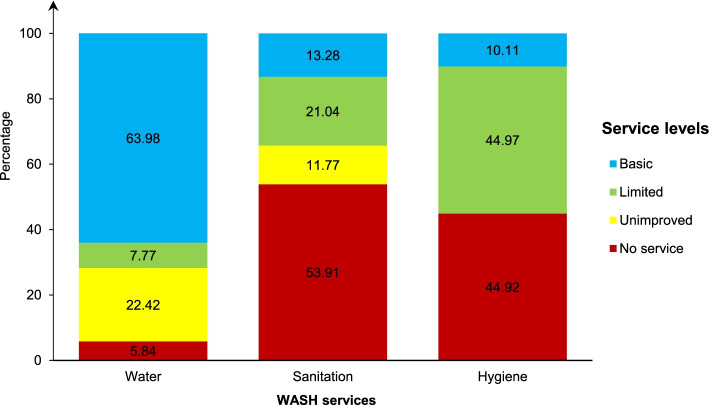
Level of household access to water, sanitation and hygiene (WASH) services in Benin, 2017–2018

Figure 1 shows the level of household access to WASH services.

#### Water

About 64% (95% CI = 61.63–66.26) of households had access to basic drinking water services versus 5.84% (95% CI = 4.70–7.23) using surface water for drinking.

**Fig. 2:**
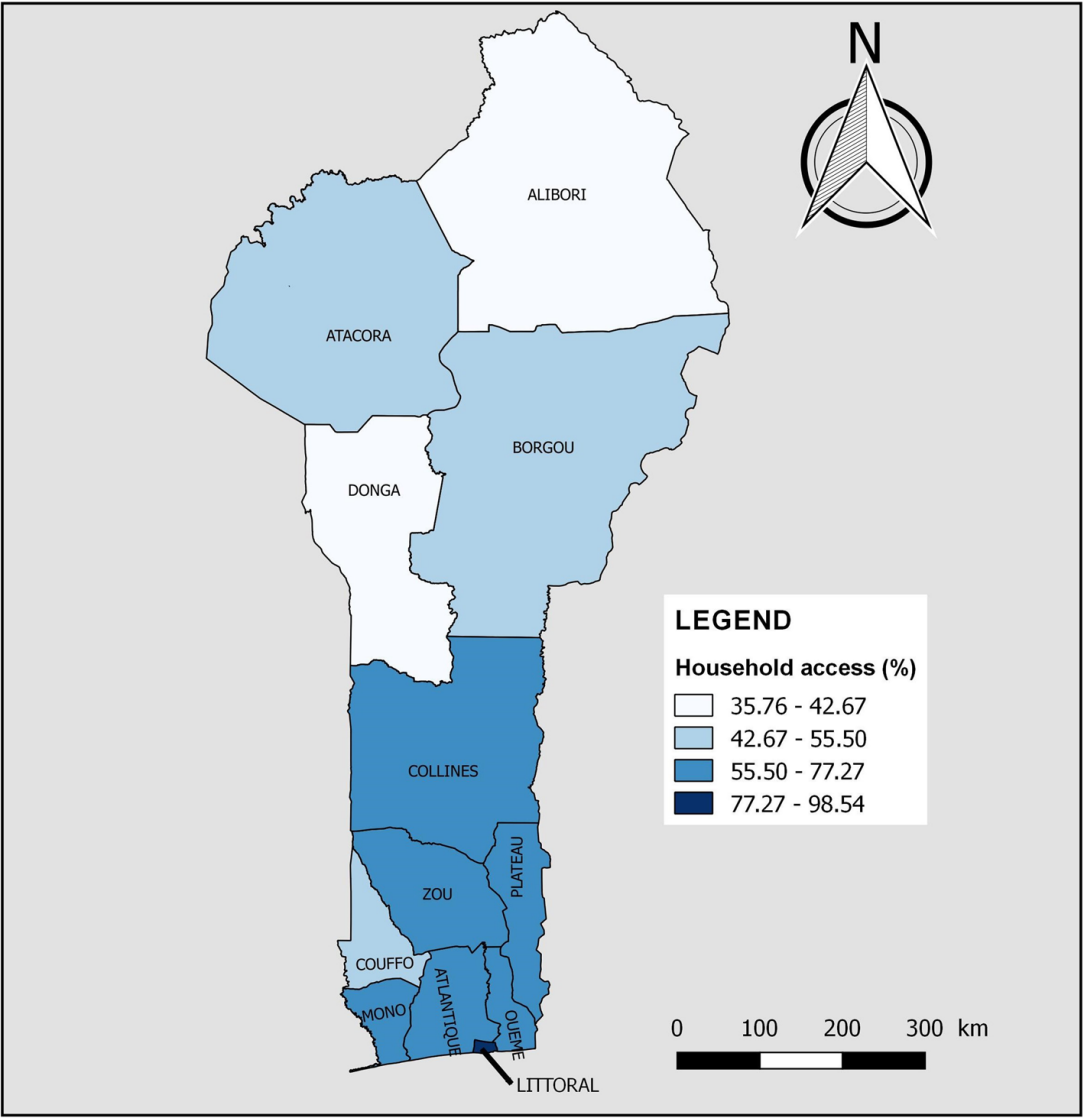
Household access to basic drinking water services by department of residence in Benin, 2017–2018

Table 4 analyses the association between independent variables and household access to individual and combined basic WASH services. Household access to basic drinking water services varied significantly with the age of the household head (*p* = 0.002). It increased significantly with the level of education of the household head (*p* < 0.001) and with the wealth index (*p* < 0.001). Furthermore, it was significantly higher in households where the head was female (68.22% vs 62.57%, *p* < 0.001) or single (66.20% vs 63.34%, *p* = 0.018). The pattern was similar for households with five or fewer people (67.78% vs 57.86%, *p* < 0.001) or without children aged 5 and under (67.62% vs 61.60%, *p* < 0.001). On the other hand, the proportion of households using basic drinking water services was significantly lower in rural areas than in urban areas (73.30% vs 56.91%, *p* < 0.001). Figure 2 shows household access to basic drinking water services by department of residence. There was a decrease in household coverage of basic drinking water services moving towards the northern departments. Littoral (98.54%), Ouémé (77.27%), Zou (72.14%), Atlantique (70.36%) and Plateau (70.11%) had the highest coverage compared to Atacora (50.78%), Donga (42.67%) and Alibori (35.76%) which had the lowest.

**Table 4:** Association between independent variables and household access to individual and combined basic WASH services in Benin, 2017–2018

Variables	Water					Sanitation					Hygiene					Combined WASH				
		**Yes**		**No**	***p***		**Yes**		**No**	***p***		**Yes**		**No**	***p***		**Yes**		**No**	***p***
	**n**	**%**	**n**	**%**		**n**	**%**	**n**	**%**		**n**	**%**	**n**	**%**		**n**	**%**	**n**	**%**	
**Age (years)**					0.002					< 0.001					0.225					0.001
< 30	1,562	63.63	893	36.37		220	8.95	2,235	91.05		238	9.72	2,216	90.28		44	1.78	2,411	98.22	
30–39	2,507	65.80	1,303	34.20		462	12.12	3,348	87.88		380	9.98	3,430	90.02		101	2.65	3,709	97.35	
40–49	1,924	64.79	1,046	35.21		451	15.19	2,519	84.81		324	10.92	2,645	89.08		107	3.59	2,863	96.41	
50–59	1,389	64.70	758	35.30		356	16.60	1,790	83.40		234	10.92	1,912	89.08		86	4.03	2,060	95.97	
≥ 60	1,675	60.36	1,100	39.64		392	14.11	2,384	85.89		254	9.14	2,522	90.86		87	3.14	2,688	96.86	
**Sex**					< 0.001					0.135					0.555					0.081
Male	6,654	62.57	3,980	37.43		1,383	13.01	9,251	86.99		1,085	10.21	9,548	89.79		336	3.16	10,298	96.84	
Female	2,403	68.22	1,119	31.78		497	14.12	3,025	85.88		346	9.81	3,177	90.19		89	2.51	3,434	97.49	
**Level of education**					< 0.001					< 0.001					< 0.001					< 0.001
No formal education	4,319	57.18	3,234	42.82		492	6.52	7,060	93.48		469	6.21	7,084	93.79		43	0.57	7,510	99.43	
Primary	2,164	66.97	1,067	33.03		427	13.21	2,805	86.79		303	9.39	2,929	90.61		55	1.71	3,177	98.29	
Secondary	1,899	73.23	694	26.77		587	22.64	2,006	77.36		401	15.44	2,193	84.56		145	5.57	2,449	94.43	
Higher	674	86.68	104	13.32		374	48.09	404	51.91		258	33.18	520	66.82		182	23.36	596	76.64	
**Marital status**					0.018					0.784					0.550					0.948
Single	2,098	66.20	1,071	33.80		426	13.45	2,743	86.55		330	10.40	2,839	89.60		94	2.98	3,074	97.02	
In couple	6,959	63.34	4,028	36.66		1,454	13.23	9,533	86.77		1,101	10.02	9,886	89.98		330	3.01	10,657	96.99	
**Wealth index**					< 0.001					< 0.001					< 0.001					< 0.001
Poorest	1,034	41.32	1,468	58.68		3	0.10	2,499	99.90		96	3.82	2,406	96.18		0	0.00	2,501	100.00	
Poorer	1,412	52.78	1,263	47.22		23	0.85	2,652	99.15		146	5.47	2,528	94.53		1	0.02	2,674	99.98	
Middle	1,653	59.06	1,145	40.94		72	2.57	2,726	97.43		179	6.41	2,619	93.59		0	0.00	2,798	100.00	
Richer	2,086	70.68	865	29.32		412	13.96	2,539	86.04		255	8.63	2,696	91.37		18	0.60	2,933	99.40	
Richest	2,873	88.93	358	11.07		1,371	42.43	1,860	57.57		755	23.37	2,476	76.63		406	12.58	2,825	87.42	
**Household size**					< 0.001					0.330					0.033					0.003
≤ 5	5,916	67.78	2,812	32.22		1,184	13.56	7,544	86.44		923	10.58	7,805	89.42		297	3.40	8,431	96.60	
> 5	3,141	57.86	2,287	42.14		697	12.83	4,731	87.17		508	9.36	4,920	90.64		128	2.36	5,300	97.64	
**Children aged 5 and under in the household**					< 0.001					< 0.001					0.008					< 0.001
No	3,785	67.62	1,813	32.38		894	15.97	4,704	84.03		624	11.14	4,975	88.86		220	3.94	5,378	96.06	
Yes	5,271	61.60	3,286	38.40		986	11.53	7,571	88.47		807	9.44	7,750	90.56		204	2.39	8,353	97.61	
**Area**					< 0.001					< 0.001					< 0.001					< 0.001
Urban	4,474	73.30	1,630	26.70		1,365	22.36	4,739	77.64		841	13.78	5,262	86.22		369	6.04	5,735	93.96	
Rural	4,583	56.91	3,470	43.09		515	6.40	7,537	93.60		590	7.32	7,463	92.68		56	0.69	7,996	99.31	
**Department**					< 0.001					< 0.001					< 0.001					< 0.001
Alibori	426	35.76	765	64.24		47	3.97	1,144	96.03		98	8.25	1,093	91.75		6	0.53	1,185	99.47	
Atacora	469	50.78	455	49.22		39	4.20	885	95.80		38	4.11	885	95.89		11	1.17	913	98.83	
Atlantique	1,385	70.36	584	29.64		355	18.02	1,614	81.98		359	18.24	1,610	81.76		126	6.38	1,843	93.62	
Borgou	788	52.63	709	47.37		131	8.72	1,367	91.28		178	11.90	1,319	88.10		30	2.03	1,467	97.97	
Collines	652	66.44	329	33.56		51	5.19	930	94.81		135	13.75	846	86.25		11	1.13	970	98.87	
Couffo	615	55.50	493	44.50		62	5.62	1,046	94.38		108	9.76	1,000	90.24		9	0.80	1,099	99.20	
Donga	316	42.67	424	57.33		40	5.35	701	94.65		41	5.52	700	94.48		2	0.26	739	99.74	
Littoral	839	98.54	12	1.46		293	34.35	559	65.65		246	28.83	606	71.17		135	15.90	716	84.10	
Mono	606	69.00	272	31.00		92	10.48	787	89.52		38	4.33	841	95.67		6	0.64	873	99.36	
Ouémé	1,261	77.27	371	22.73		328	20.07	1,305	79.93		121	7.44	1,511	92.56		60	3.65	1,573	96.35	
Plateau	690	70.11	294	29.89		125	12.68	859	87.32		15	1.50	969	98.50		5	0.56	979	99.44	
Zou	1,009	72.14	390	27.86		319	22.81	1,079	77.19		54	3.85	1,345	96.15		24	1.69	1,375	98.31

#### Sanitation

In 53.91% (95% CI = 51.35–56.44) of the households, members practiced open defecation. Basic sanitation services were reported in 13.28% (95% CI = 12.10–14.57) of households, respectively.

**Fig. 3:**
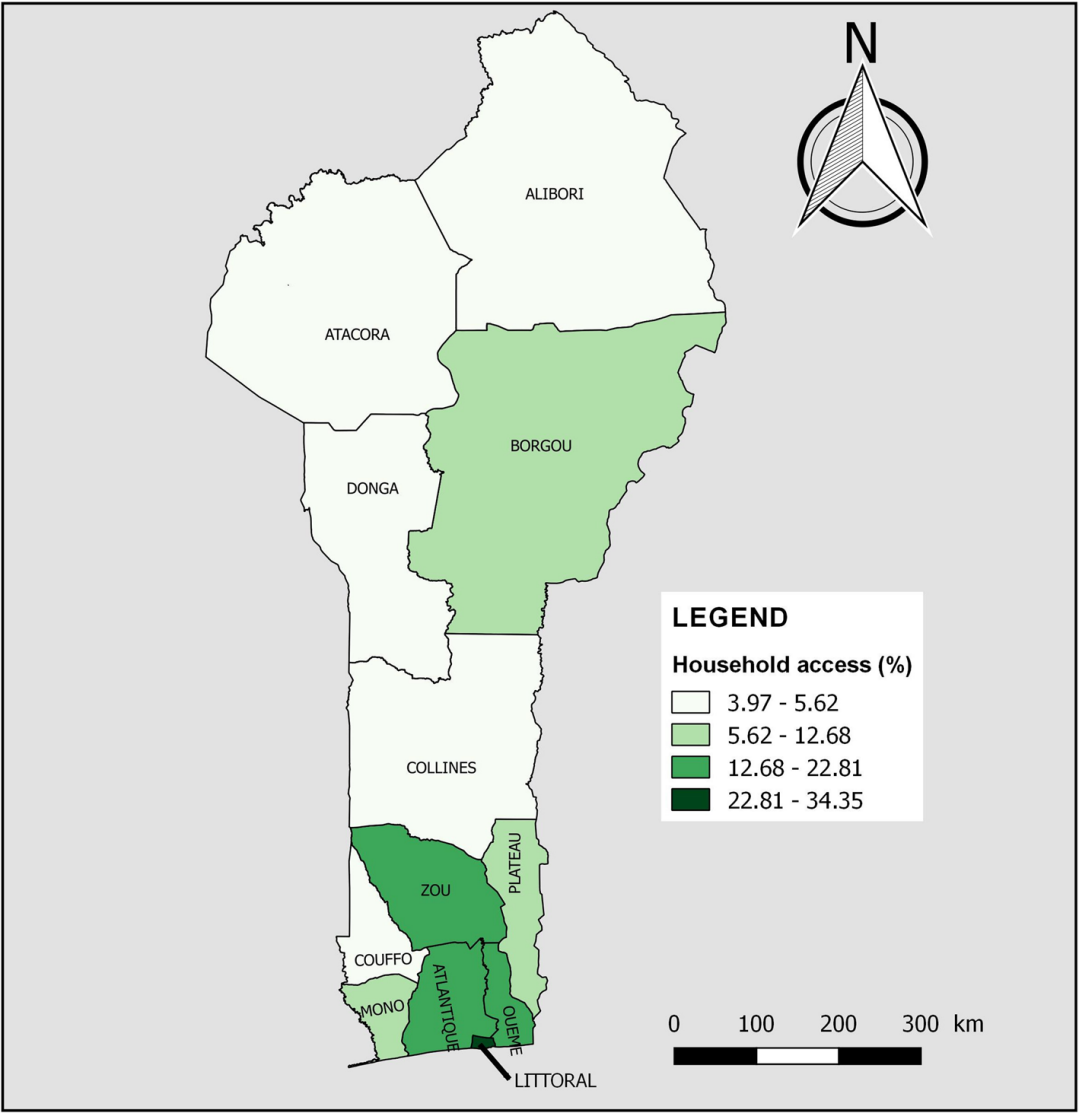
Household access to basic sanitation services by department of residence in Benin, 2017–2018

According to Table 4, household coverage of basic sanitation services showed significant differences by age (*p* < 0.001) and level of education (*p* < 0.001) of the household head, and by wealth index (p < 0.001). Also, household access to improved non-shared (basic) sanitation facilities was significantly higher in urban areas (22.36% vs 6.40%, *p* < 0.001) and in households without children aged 5 and under (15.97% vs 11.53%, *p* < 0.001). Figure 3 shows household access to basic sanitation services by department of residence. The departments in the South and Centre, notably Littoral (34.35%), Zou (22.81%), Ouémé (20.07%) and Atlantique (18.02%), had the highest coverage, unlike Atacora (4.20%) and Alibori (3.97%) in the North (Fig. 3).

#### Hygiene

Basic handwashing facilities were in 10.11% (95% CI = 9.19–11.11) of households. In contrast, 44.92% (95% CI = 42.72–47.14) had no handwashing facilities.

**Fig. 4:**
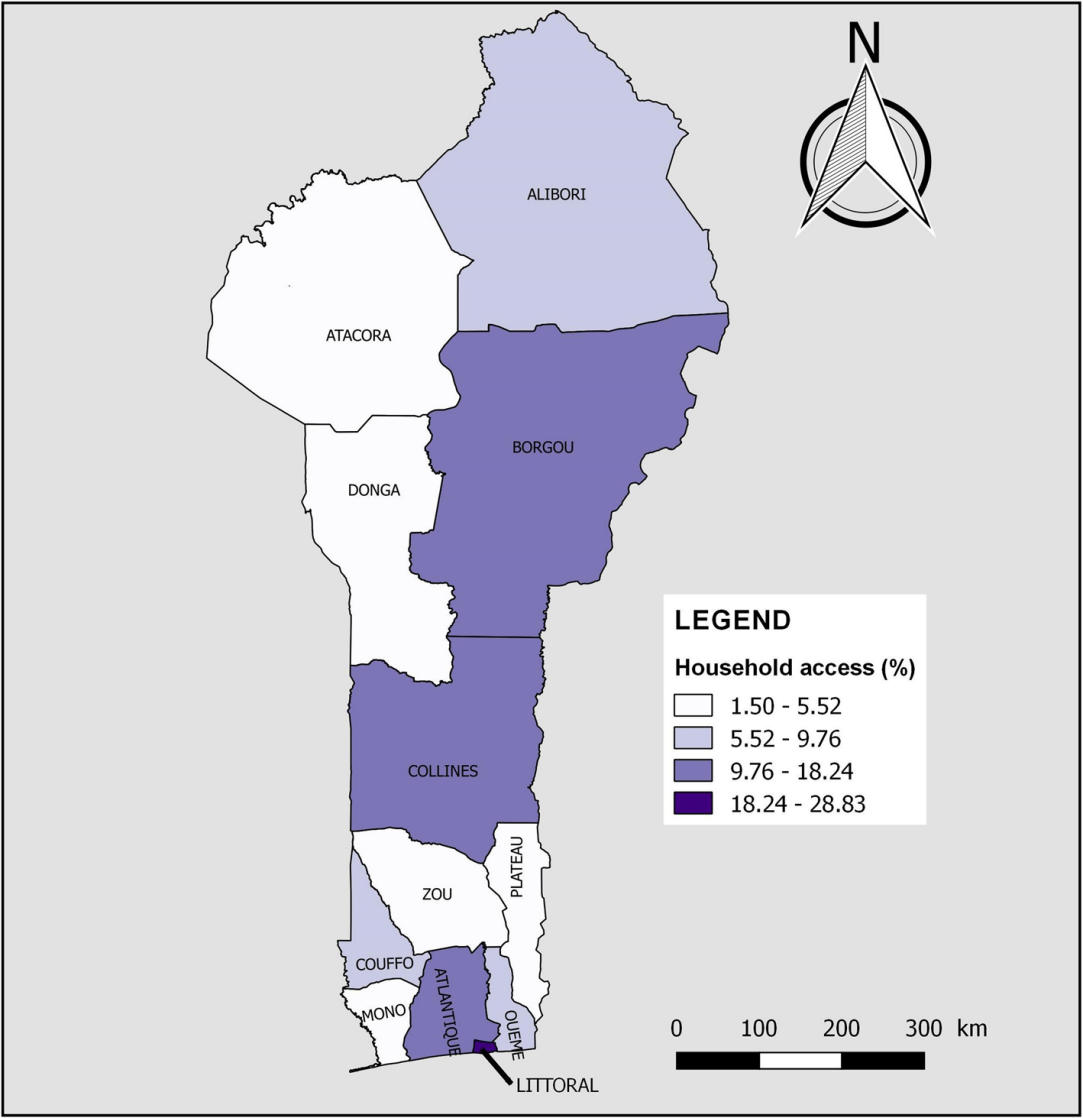
Household access to basic hygiene services by department of residence in Benin, 2017–2018

According to Table 4, the availability of handwashing facilities with soap and water increased significantly with the level of education of the household head (*p* < 0.001). About 24% of the richest households had access to basic handwashing facilities, whereas fewer than 10% of households in the other four wealth quintiles had access to such facilities (*p* < 0.001). The availability of basic handwashing facilities was significantly higher in households with five or fewer people (10.58% vs 9.36%, *p* = 0.033), with no children aged 5 and under (11.14% vs 9.44%, *p* = 0.008) and living in urban areas (13.78% vs 7.32%, p < 0.001). Figure 4 shows household access to basic hygiene services by department of residence. Household access to basic handwashing facilities was highest in Littoral (28.83%), Atlantique (18.24%), Collines (13.75%) and Borgou (11.90%). In the other departments, less than one household in ten had such facilities.

#### Combined WASH

**Fig. 5:**
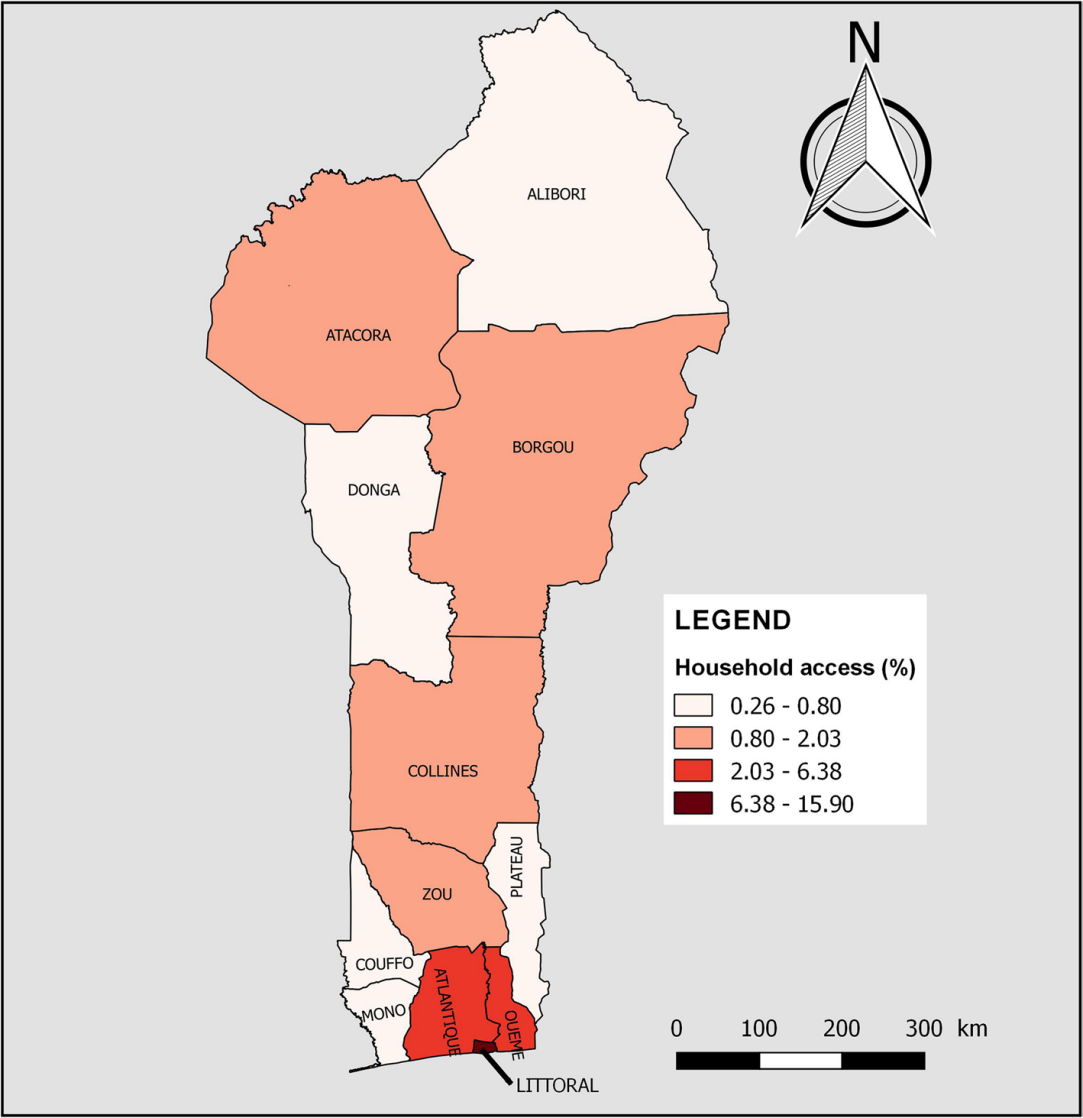
Household access to combined basic WASH services by department of residence in Benin, 2017–2018

About 3% (95% CI = 2,53–3,56) of households had access to combined basic WASH services. According to Table 4, household access to combined basic WASH services varied significantly by age (*p* < 0.001) and level of education of the household head (*p* < 0.001). It was also higher in households with five or fewer people (3.40% vs 2.36%, *p* = 0.003), without children aged 5 and under (3.94% vs 2.39%, *p* < 0.001) and those living in urban areas (6.04% vs 0.69%, *p* < 0.001). No poorest or middle households had access. However, 12.58%, 0.60% and 0.02% of the poorer, richer and richest household had access to combined basic WASH services, respectively (*p* < 0.001). Figure 5 shows household access to combined basic WASH services by department of residence. In Couffo (0.80%), Mono (0.64%), Plateau (0.56%), Alibori (0.53%) and Donga (0.26%), less than one in 100 households had access to combined basic WASH facilities.

### Factors associated with WASH services

Table 5 presents the results of the multivariate analysis and highlights the factors associated with household access to individual and combined basic WASH services.

**Table 5:** Factors associated with household access to individual and combined basic WASH services, 2017–2018

Variables	Water			Sanitation			Hygiene			Combined WASH		
	**aOR**	**95% CI**	***p***	**aOR**	**95% CI**	***p***	**aOR**	**95% CI**	***p***	**aOR**	**95% CI**	***p***
**Age (years)**												
< 30	Ref			Ref			Ref			Ref		
30–39	1.20	1.05–1.38	0.008	1.71	1.37–2.13	< 0.001	1.16	0.94–1.43	0.155	1.88	1.2–2.94	0.006
40–49	1.19	1.04–1.38	0.014	3.33	2.68–4.13	< 0.001	1.49	1.22–1.83	< 0.001	4.15	2.77–6.21	< 0.001
50–59	1.17	1.01–1.35	0.035	3.78	2.94–4.84	< 0.001	1.51	1.22–1.87	< 0.001	4.26	2.9–6.26	< 0.001
≥ 60	1.06	0.92–1.22	0.431	4.80	3.76–6.12	< 0.001	1.65	1.32–2.06	< 0.001	6.02	3.95–9.18	< 0.001
**Sex**												
Male	Ref			Ref								
Female	1.13	1.01–1.25	0.031	1.32	1.14–1.53	< 0.001						
**Level of education**												
No formal education				Ref			Ref			Ref		
Primary				1.09	0.91–1.30	0.336	1.22	1.01–1.46	0.040	1.23	0.81–1.87	0.329
Secondary				1.65	1.37–1.98	< 0.001	1.71	1.42–2.05	< 0.001	2.92	1.99–4.27	< 0.001
Higher				3.54	2.73–4.59	< 0.001	3.18	2.50–4.04	< 0.001	9.84	6.55–14.77	< 0.001
**Wealth index**												
Poorest	Ref			Ref			Ref			Ref		
Poorer	1.48	1.26–1.74	< 0.001	7.61	1.48–39.28	0.015	1.40	1.02–1.93	0.038			
Middle	1.85	1.54–2.22	< 0.001	22.13	4.55–107.55	< 0.001	1.61	1.19–2.19	0.002			
Richer	2.80	2.27–3.45	< 0.001	143.28	29.90–686.56	< 0.001	2.18	1.58–3.01	< 0.001	19.27	2.63–141.13	0.004
Richest	7.06	5.38–9.27	< 0.001	651.82	136.20–3,119.53	< 0.001	4.93	3.54–6.88	< 0.001	380.23	55.99–2,581.98	< 0.001
**Household size**												
≤ 5	1.15	1.04–1.28	0.007									
> 5	Ref											
**Department**												
Alibori	Ref			1.86	1.12–3.10	0.017	8.34	4.19–16.60	< 0.001	5.27	1.10–25.16	0.037
Atacora	2.04	1.34–3.12	0.001	1.51	0.82–2.78	0.181	3.45	1.60–7.43	0.002	6.89	1.58–29.96	0.010
Atlantique	2.51	1.54–4.09	< 0.001	2.13	1.43–3.18	< 0.001	10.10	5.06–20.15	< 0.001	12.25	2.91–51.64	0.001
Borgou	1.74	1.16–2.61	0.007	1.81	1.10–2.97	0.019	9.04	4.73–17.28	< 0.001	6.43	1.57–26.30	0.010
Collines	2.82	1.76–4.51	< 0.001	Ref			10.67	5.50–20.69	< 0.001	4.46	0.83–24.12	0.082
Couffo	1.92	1.15–3.22	0.013	1.65	1.00–2.75	0.052	7.96	4.00–15.85	< 0.001	4.20	0.83–21.22	0.082
Donga	1.01	0.63–1.64	0.956	1.04	0.56–1.93	0.912	3.72	1.64–8.44	0.002	Ref		
Littoral	32.13	14.23–72.56	< 0.001	1.49	0.99–2.23	0.054	9.10	4.62–17.92	< 0.001	10.77	2.66–43.62	0.001
Mono	3.12	1.79–5.43	< 0.001	1.92	1.22–3.02	0.005	2.50	1.02–6.11	0.044	1.65	0.29–9.28	0.568
Ouémé	3.28	2.05–5.24	< 0.001	2.05	1.35–3.11	0.001	3.20	1.60–6.38	0.001	5.73	1.41–23.29	0.015
Plateau	3.48	2.17–5.58	< 0.001	3.60	2.23–5.81	< 0.001	Ref			2.57	0.41–16.05	0.311
Zou	3.27	2.02–5.27	< 0.001	6.44	4.04–10.29	< 0.001	2.19	1.08–4.42	0.029	4.81	1.15–20.20	0.032

*aOR* Adjusted Odd Ratio, *Ref* Reference category

For identifying predictors of combined basic WASH services, the categories 'Poorest' and ' Poorer' of the 'Wealth Index' were put together, as well as 'Middle' and 'Richer'

#### Water

Factors associated with household access to basic water facilities were age and sex of the household head, and size, wealth index and department of the household. The odds of having access to basic water facilities was significantly higher in households whose heads were aged 30–39 (aOR = 1.20, 95% CI = 1.05–1.38), compared to those whose heads were under 30. Compared to male-headed households, female-headed households were 1.13 times (aOR = 1.13, 95% CI = 1.01–1.25) more likely to have access to basic drinking water services. Also, the odds of having access to basic drinking water services increased significantly with the wealth index. Compared to the poorest households, the richest households were 7.06 times (aOR = 7.06, 95% CI = 5.38–9.27) more likely to have access. Households with five or fewer people were 1.15 times (aOR = 1.15, 95% CI = 1.04–1.28) more likely to have access to basic water facilities compared to households with more than five people. Compared to households in Alibori, those in Littoral (aOR = 32.13, 95% CI = 14.23—72.56) had much higher odds of basic water service coverage.

#### Sanitation

Factors associated with household access to basic sanitation facilities were age, sex and level of education of the household head, and wealth index and department of the household. The likelihood of a household with a head aged 60 and over having access to basic sanitation facilities was multiplied by 4.80 (aOR = 4.80, 95% CI = 3.76–6.12) compared to a household headed by a person under 30 years old. Female-headed households (aOR = 1.32, 95% CI = 1.14–1.53) were more likely to have access to basic sanitation facilities. Households with higher educated heads were 3.54 times (aOR = 3.54, 95% CI = 2.73–4.59) more likely to have access to basic sanitation facilities compared to households with heads who had no formal education. The richest households (aOR = 651.82, 95% CI = 136.20–3,119.53) were more likely to have access to basic sanitation facilities than the poorest households. Furthermore, compared to Collines, Zou (aOR = 6.44, 95% CI = 4.04–10.29) were associated with significantly higher odds of access to basic sanitation services.

#### Hygiene

Factors associated with household access to basic handwashing facilities were age and level of education of the household head, and wealth index and department of the household. Thus, households with a head aged 60 years and over were 1.65 times (aOR = 1.65, 95% CI = 1.32–2.06) more likely to have handwashing facilities with soap and water compared to households with heads under 30 years old. Households whose heads had a higher level of education were 3.18 times (aOR = 3.18, 95% CI = 2.50–4.04) more likely to have access to basic hygiene services than households headed by people with no formal education. The richest households were 4.93 times (aOR = 4.93, 95% CI = 3.54–6.88) more likely to have basic handwashing facilities than the poorest households. Compared to Plateau, the other departments were associated with significantly higher odds of access to basic hygiene services (p < 0.05). Households in the Atlantique, Collines and Littoral were 10.10 (aOR = 10.10, 95% CI = 5.06–20.15), 10.67 (aOR = 10.67, 95% CI = 5.50–20.69) and 9.10 (aOR = 9.10, 95% CI = 4.62–17.92) times more likely to have access to basic handwashing facilities, respectively.

#### Combined WASH

Factors associated with combined basic WASH services were age and level of education of the household head, and wealth index and department of residence of the household. Households with heads aged 60 years and above and with higher education levels were 6.02 (aOR = 6.02, 95% CI = 3.95–9.18) and 9.84 (aOR = 9.84, 95% CI = 6.55–14.77) times more likely to have access to combined basic WASH services, respectively. Also, the richest households (aOR = 380.23, 95% CI = 55.99–2,581.98) were more likely to have access to combined basic WASH services than the poorest/poorer. The odds of a household having access to combined basic WASH services were significantly higher in Atlantique (aOR = 12.25, 95% CI = 2.91–51.64) and Littoral (aOR = 10.77, 95% CI = 2.66–43.62), compared to Donga.

## Discussion

This study aimed to provide an overview of household access to WASH facilities using nationally representative data. The study estimated the proportion of households using basic WASH services and identified predictors of access to these facilities. The use of nationally representative data, which can improve the generalisation of results, is one of the strengths of this study.

About 6% of households used water from rivers, dams, lakes, ponds, streams, canals or irrigation canals. Based on the results of previous DHS, there is a downward trend in the proportion of households using surface water for drinking (12.1% in 2001, 9.9% in 2006, 3.6% in 2011–2012 and 5.84% in 2017–2018) [33, 39, 40]. According to the results of this study, 63.98% and 7.77% of households used basic and limited drinking water facilities, respectively. It indicates that 71.75% of households use improved drinking water facilities. Hence, the proportion of households using such facilities increased by 4% between 2001 and 2017–2018, from 66.50% to 71.75% [33, 39, 40]. By comparison, the proportion of households with access to improved drinking water sources found in other African countries and Asia was higher than that noted here. Indeed, a percentage ranging from 68.5% to 97.6% of households using water from an improved source was recorded in Ethiopia [21, 22], Ghana [23], Malaysia [24], Eswatini [27] and Vietnam [30]. Regarding household access to basic drinking water services, a study in Bangladesh in 2021 reported a proportion of 99.5% compared to 63.98% in this study [25]. This proportion is still higher than that estimated for sub-Saharan Africa (53.6%) in 2017 [41].

More than half of the households had no access to sanitation facilities or toilets and practised open defecation. There is evidence of a gradual decrease in the proportion of households practising open defecation: from 67% in 2001 to 61.7% in 2006, then 54.2% in 2011–2012 and finally 53.91% in 2017–2018 [33, 39, 40]. This decrease can be attributed to various interventions in Benin to reduce the prevalence of open defecation [14–17]. Between 2014 and 2017, the CTPS approach resulted in 2,724 localities achieving open defecation free status [16]. Nevertheless, there is a need to strengthen initiatives to combat open defecation. In sub-Saharan Africa, the overall prevalence of open defecation (53.91%) is about half that observed in this study [41]. It ranged from 12.02% in East Africa to 31.10% in West Africa [41]. Furthermore, 21.04% and 13.28% of the surveyed households used limited and basic sanitation services, respectively. Thus, 34.32% had access to improved sanitation facilities. Studies in Ethiopia, Ghana, Vietnam and Afghanistan found percentages ranging from 12 to 85.7% [22, 23, 28–30]. The results of a study in Malaysia indicated that this country has almost achieved universal coverage of improved sanitation facilities [24]. The present study found that only 13.28% of households use improved non-shared (basic) sanitation facilities. However, this proportion has increased significantly from 2001 to 2017–2018 (3% in 2001, 7% in 2006, 15.2% in 2011–2012, and 13.28% in 2017–2018) [33, 39, 40]. In Bangladesh, the proportion of households using basic sanitation services is more than four times higher than the proportion we found [25].

The proportion of households without handwashing facilities has decreased substantially over the past two decades (96.1% in 2001, 94.1% in 2006, 68.9% in 2011–2012 and 44.92% in 2017–2018) [33, 39, 40]. However, only 10.11% of households had basic handwashing facilities. A study in 2021 reported a proportion of 56.3% [25].

Approximately 3% of households had access to combined basic WASH services, compared to 40.2% in Bangladesh in 2021 [25]. In sub-Saharan Africa, household-level access to combined basic WASH services ranges from 0.8% in Liberia to 22.6% in Namibia, with a regional average of only 4.2% [41].

The results of this study suggest that households with heads aged 30 and over were more likely to have access to individual and combined basic WASH services. The older the age of the household head, the more likely households were to have access to these services. One possible explanation is that individuals aged 30 and over, in the majority of cases, are in the active economic group and are more likely to install basic WASH facilities, especially when they own their homes [28]. Also, the low level of financial resources available to some young people, especially those under 30, may lead them to opt for cheap homes (especially rented ones), which may not have basic WASH facilities. Also, some authors suggest that as people age, they make choices or adopt behaviours that improve their quality of life [23]. The findings of some studies in Ethiopia and Ghana are consistent with the current one [21, 23, 28]. They indicated that households headed by older people were more likely to have access to improved water and sanitation facilities [21, 23, 28]. In contrast, in Eswatini, the age of the household head was negatively associated with the use of water from an improved source [27]. The older the household head, the less likely the household was to have access to improved water sources. According to the authors, the majority of older subjects (i.e. 35 years and older) in their sample came from poor households and mainly from rural areas and therefore could not afford improved drinking water sources [27].

The odds for access to basic water and sanitation facilities were significantly higher among female-headed households. Some studies reported similar results [22, 23]. Some authors suggest that, compared to men, most women in sub-Saharan Africa have greater responsibilities within the household, associated with high water use [23]. To reduce the burden of fetching water in remote locations, women heads of household ensure that their families have good access to water and sanitation facilities [23]. Findings in the opposite direction were found by other studies; i.e. male-headed households were more likely to have access to improved drinking water and sanitation facilities [27, 28]. Concerning access to water, one of the reasons given was that households with low coverage of improved water sources are located in rural areas, where most female-headed households are found [27].

A positive relationship was found between the level of education of the household head and access to basic sanitation and hygiene services. The higher the education level of the household head, the more likely the household was to be covered by these services. The same relation is noted for access to combined basic WASH services. These results are consistent with findings from other studies [22, 23, 25, 28, 30]. The level of education is an essential social determinant of health that influences, most notably, the ability to make better decisions about the health of household members [42]. In the present study, the more educated household heads would be more aware of the benefits of improved and basic WASH conditions. Since it is not possible to improve the level of education of household heads at a late point in their lives, some authors have suggested an approach based on large-scale promotional campaigns focusing on people's access to WASH services [25]. Some studies have shown the beneficial effects of mass awareness and promotion interventions on increasing access to WASH facilities [43, 44].

The findings of this study suggest that the better the wealth status of households, the more likely they are to be covered by individual and combined basic WASH services. Indeed, access to these facilities increased significantly from the second wealth quintile (poorer) onwards, reaching its highest level in the last quintile (richest). Other studies supported these results [22–25, 27, 28, 30]. The richest households can afford the costs associated with connection to the National Water Company (SONEB, *Société Nationale des Eaux du Benin* in French), and the installation of improved private toilets and functional handwashing facilities in the home. These expenditures may seem high for the poorest households who face other constraints and daily expenses. Specifically for this study, there appears to be a disproportionate difference in the odds of access to individual and combined basic WASH facilities between the richest and poorest households. In recent years, interventions to improve access to WASH have focused on the poorest people [14]. The results of this study show the relevance of this strategy but indicate that much more needs to be done. Providing financial assistance to the poorest populations to enable them to acquire adequate WASH equipment is an option that should be explored. In this perspective, the path of microfinance, which has the potential to offer more flexibility to the poorest, deserves to be taken. Microfinance has evolved considerably over the past decades, from micro-credit for the poorest to a wide range of financial and non-financial services and products targeted to the needs of poor men and women [45]. Integrating population-based WASH products with microfinance has been tested in some settings, with interesting results [46–48].

This study found a negative association between an increase in the number of members in the household and access to basic drinking water services. This result is in line with the findings of a study in Eswatini in 2020 in which the authors advocated for enhanced promotion of family planning products [27]. In contrast, a positive association between household size and access to basic WASH facilities was found in Bangladesh in 2021 [25]. According to the authors, regardless of their economic status, larger families may spend more on basic WASH facilities [25]. In the Beninese context, where some studies show a positive relationship between poverty and household size [49, 50], a high number of people in the household is associated with high expenditure on water consumption. This could explain why some households choose to use unimproved facilities to limit the costs of using basic water facilities.

We found that the proportion of households with access to individual and combined basic WASH services was higher in urban than in rural areas. However, all other things being equal, area of residence was not a factor associated with household access to WASH services.

Compared to basic sanitation and hygiene facilities, basic drinking water services were the most available at the household level, with the Littoral reaching universal coverage. Interventions need to be strengthened so that everyone has effective access. As regards basic sanitation and hygiene services, the coverage observed remains relatively low, even in the Littoral, although it has the highest level of access. Specifically for the country, this study found that all other things being equal, households in Alibori, Collines, Plateau and Donga were the least likely to have access to individual and combined basic WASH services. They should therefore be the focus of special attention.

The main limitation of this study is that it is a cross-sectional study, which does not allow a causal relationship to be established with certainty between the identified predictors and the outcomes.

## Conclusion

In 24, despite the progress made, there is still a lack of coverage of households with basic WASH facilities. Only 3% of households in Benin had the three main components of WASH facilities at a basic level. The study also identified predictors of household access to individual and combined basic WASH. Overall, the richest households and few, and those headed by people over 30 years old, female and with higher levels of education, were the most likely to have access to individual and combined basic WASH. In addition, disparities based on the department of residence were observed. This reflects the multifactorial reality of the issue of people's access to appropriate WASH services. Consequently, isolated or single-factor interventions can only lead to limited results. This will require better integration of the various interventions carried out as well as better concertation and coordination between the actors. The CLTS approach developed by UNICEF, which aims to end open defecation, is a good example. On the basis of the results, the following suggestions were made: implement large-scale promotion campaigns focusing on people's access to WASH services, strengthen poverty fighting at national and local levels and provide financial support to the poorest people, promote family planning and take into account regional disparities. Future studies can examine the impact of additional factors on household access to WASH services that have not been studied here.

## Data Availability

The data used in this study can be obtained by sending a request via the DHS Program website to https://dhsprogram.com/data/dataset/Benin_Standard-DHS_2017.cfm?flag=0

## Abbreviations

aOR: Adjusted Odd Ratio
CI: Confidence Interval
CNERS: National Committee on Health Research Ethics (*Comité National d’Ethique pour la Recherche en Santé*)
CNS: National Statistical Council (*Conseil National de la Statistique*)
cOR: Crude Odd Ratio
DALYs: Disability-Adjusted Life Years
CLTS: Community-Led Total Sanitation
DHS: Demographic and Health Survey
EA: Enumeration Area
INStaD: National Institute of Statistics and Demography (*Institut National de la Statistique et de la Démographie*)
JMP: WHO/UNICEF Joint Monitoring Programme
PND: National Development Plan (*Plan National de Développement*)
PNDS: Health Development Plan (*Plan National de Développement Sanitaire*)
PPS: Probability Proportional to the Size
PSU: Primary Sample Units
RGPH: General Census of Population and Housing (*Recensement Général de la Population et de l'Habitation*)
SDG: Sustainable Development Goals
SNPHAB: National Strategy for the Promotion of Hygiene and Basic Sanitation (*Stratégie Nationale de Promotion de l’Hygiène et de l’Assainissement de Base*)
SONEB: Benin National Water Company (*Société Nationale des Eaux du Benin*)
UNGA: United Nations General Assembly
UNICEF: United Nations International Children's Fund
USAID: United States Agency for International Development
WASH: Water, Sanitation and Hygiene
WHO: World Health Organization
